# Engineering a Vascularized 3D Hybrid System to Model Tumor-Stroma Interactions in Breast Cancer

**DOI:** 10.3389/fbioe.2021.647031

**Published:** 2021-03-11

**Authors:** Filipa C. Teixeira, Sara Chaves, Ana Luísa Torres, Cristina C. Barrias, Sílvia J. Bidarra

**Affiliations:** ^1^i3S – Instituto de Inovação e Investigação em Saúde, Porto, Portugal; ^2^INEB – Instituto de Engenharia Biomédica, Universidade do Porto, Porto, Portugal; ^3^ICBAS – Instituto de Ciências Biomédicas Abel Salazar, Universidade do Porto, Porto, Portugal

**Keywords:** hydrogel, alginate, vascularized stroma, outgrowth endothelial cells, angiogenesis, tissue engineering, organoid

## Abstract

The stromal microenvironment of breast tumors, namely the vasculature, has a key role in tumor development and metastatic spread. Tumor angiogenesis is a coordinated process, requiring the cooperation of cancer cells, stromal cells, such as fibroblasts and endothelial cells, secreted factors and the extracellular matrix (ECM). *In vitro* models capable of capturing such complex environment are still scarce, but are pivotal to improve success rates in drug development and screening. To address this challenge, we developed a hybrid alginate-based 3D system, combining hydrogel-embedded mammary epithelial cells (parenchymal compartment) with a porous scaffold co-seeded with fibroblasts and endothelial cells (vascularized stromal compartment). For the stromal compartment, we used porous alginate scaffolds produced by freeze-drying with particle leaching, a simple, low-cost and non-toxic approach that provided storable ready-to-use scaffolds fitting the wells of standard 96-well plates. Co-seeded endothelial cells and fibroblasts were able to adhere to the surface, spread and organize into tubular-like structures. For the parenchymal compartment, a designed alginate gel precursor solution load with mammary epithelial cells was added to the pores of pre-vascularized scaffolds, forming a hydrogel *in situ* by ionic crosslinking. The 3D hybrid system supports epithelial morphogenesis in organoids/tumoroids and endothelial tubulogenesis, allowing heterotypic cell-cell and cell-ECM interactions, while presenting excellent experimental tractability for whole-mount confocal microscopy, histology and mild cell recovery for down-stream analysis. It thus provides a unique 3D *in vitro* platform to dissect epithelial-stromal interactions and tumor angiogenesis, which may assist in the development of selective and more effective anticancer therapies.

## Introduction

Breast cancer is the leading cause of cancer death in women worldwide, accounting for approximately 7% of the nearly 10 millions of cancer deaths in 2018, according to the International Agency for Research on Cancer. Despite an overwhelming amount of pre-clinical research on breast cancer models and chemotherapeutic strategies, the prospect of a an oncology drug being approved after completing Phase 1 trials was only ca. 5% during the 2006–2015 period (Thomas et al., 2016). This low efficiency is partly due to the inherently low ability of current preclinical cancer models to faithfully mimic the patients native breast cancer microenvironment. Significantly, while it is nowadays clear that the supportive tumoral niche plays a key role in cancer progression and drug resistance, this is often overlooked in cancer modeling (Langhans, 2018). Thus, the design of new models that better recapitulate *in vivo* tumor biology and microenvironmental features is pivotal to improve success rates in drug development and screening. It is now well-accepted that three-dimensional (3D) *in vitro* systems better emulate the *in vivo* environment than the traditional two-dimensional (2D) monolayer cultures (Pape et al., 2019). Due to their superior biological relevance and consequently higher predictive value for the therapeutic outcome, 3D cell cultures are becoming more prominent in drug discovery. In addition, 3D cell culture models using human cells can circumvent drawbacks of rodent models that, aside from the high cost and ethical considerations, are often non-representative of human-specific conditions.

Breast cancer is a heterogeneous disease that differs greatly not only among patients, but also within each individual tumor, which may explain the variability regarding therapeutic responses and disease progression (Coates et al., 2015; Baliu-Pique et al., 2020). 3D organotypic models represent powerful tools to replicate such heterogeneity, including tumor-stroma interactions, being central for comprehending cancer-related mechanisms and drug response. Organoids, self-organizing multicellular structures that mimic essential features of real tissues/organs, have emerged as physiologically relevant *in vitro* models to study cancer (Drost and Clevers, 2018). Still, they are commonly assembled in extracellular matrix (ECM)-derived 3D matrices, such as type I collagen or Matrigel^TM^ which possess poorly tunable biochemical/mechanical properties, high batch-to-batch variability and intrinsic bioactivity, making it difficult to perform mechanistic studies and compare results between different laboratories or even different experiments. Biomaterial-based platforms traditionally associated with tissue engineering approaches have been translated into cancer research, creating improved models to study tumor biology (Gill and West, 2014; Bray et al., 2015; Bidarra et al., 2016; Taubenberger et al., 2016; Belgodere et al., 2018; Papalazarou et al., 2018). In particular, *in vitro* models based on ECM-mimetic hydrogels exhibit great potential as matrices for 3D cell culture and morphogenesis (Park et al., 2017; Bidarra and Barrias, 2019; Monteiro et al., 2020; Torres et al., 2020). Among these, ultra-pure alginate hydrogels present key advantages, including: (i) low batch-to-batch variability; (ii) well defined and xeno-free composition; (iii) precisely customizable biochemical/physical properties; (iv) transparency for routine monitoring of cell morphology and growth along culture by optical microscopy; and (iv) reversible hydrogel formation by ionic crosslinking, allowing hydrogel dissolution with chelating agents for mild cell recovery after culture (Bidarra and Barrias, 2019; Araujo et al., 2020; Campiglio et al., 2020). We have previously shown that bioengineered alginate hydrogels support mammary epithelial cell morphogenesis into organoids that recapitulate histological and functional features of the mammary gland, the site from which breast cancers emerge (Bidarra et al., 2016; Barros da Silva et al., 2020).

In addition to epithelial cells, breast tumor niches also include a vascularized stromal compartment that plays a critical role in cancer progression and drug resistance, but *in vitro* models reflecting such complex environment are scarce. In particular, the proper modeling of endothelial cells (EC) recruitment and tumoroid neovascularization dynamics can greatly improve our understanding of tumor-driven angiogenesis, which is critical for tumor survival. Still, attempts to mimic *in vitro* tumor-associated vasculature by simply co-culturing cancer cells with EC have shown limited success so far. For EC to be able to migrate and organize into tubular-like structures, alongside with organoid/tumoroid development, an engineered angiogenesis-promoting microenvironment must include an ECM-like 3D scaffold rationally designed to simultaneously support both processes (Grebenyuk and Ranga, 2019).

**FIGURE 1:**
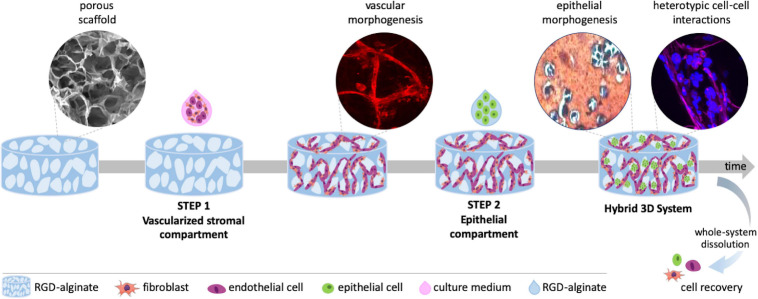
Schematic representation of the hybrid 3D system, combining a vascularized stromal with a parenchymal epithelial compartment. Alginate porous 3D scaffolds are prepared by freeze-drying and particle leaching. In step 1, outgrowth endothelial cells (OEC) and fibroblasts are co-seeded on the scaffold and tubular-like endothelial structures are formed. In step 2, epithelial cells suspended in alginate gel-precursor solution are added to the pre-vascularized scaffold for *in situ* hydrogel formation. Epithelial organoids are formed within pores in close contact with tubular-like structures, fibroblasts and their ECM, establishing heterotypic cell-cell and cell-matrix interactions. The 3D system can be imaged by confocal microscopy, processed for histology or easily dissolved for cell recovery and downstream analysis.

To address this challenge, we developed a hybrid alginate-based 3D system, combining a porous scaffold co-seeded with fibroblasts and EC (step 1: vascularized stromal compartment) with gel-embedded mammary epithelial cells (step 2: parenchymal compartment) (Figure 1). The system supports endothelial tubulogenesis and epithelial morphogenesis into prototypical mammary organoids and tumoroid-like structures, allowing both direct and indirect heterotypic cell-cell and cell-ECM communication. Importantly, it presents excellent experimental tractability for multiple and complementary analytical techniques. Thus, it provides a unique tool to dissect epithelial-stromal interactions and tumor angiogenesis toward the development of anticancer therapies.

## Materials and Methods

### Synthesis of RGD-Grafted Alginate

Ultrapure (UP) sodium alginate (PRONOVA UP LVG, Novamatrix, FMC Biopolymers) with an average molecular weight of 190 kDa and a high content of guluronic acid (≈70%) was used to produce the 3D alginate sponges and for cell embedding. Covalent grafting of the oligopeptidic RGD sequence [(Glycine)4-Arginine-Glycine-Aspartic acid-Serine-Proline, GGGGRGDSP, Peptide International] to alginate was performed by aqueous carbodiimde chemistry, as described previously (Bidarra et al., 2011b; Fonseca et al., 2011). The amount of grafted RGD was quantified using the BCA Protein Assay (Pierce). Briefly, samples (1 wt.% RGD-grafted alginate) were incubated in Bicinchoninic acid (BCA) reagent for 60 min at 37°C in the dark and the absorbance was read at 562 nm in a microplate reader (Biotek Synergy MX). A set of RGD solutions (0 to 1 mg/ml in 1 wt.% HMW0) was used as standards to produce a calibration curve.

### Preparation and Characterization of RGD-Alginate Scaffolds

Alginate porous scaffolds were prepared combining the freeze drying with particle leaching technique, using sodium chloride (NaCl) as porogen. NaCl particles were sieved to a particle size range of 150–250 μm. RGD-alginate gel precursor solutions were homogeneously mixed with a suspension of calcium carbonate in MilliQ water at a calcium carbonate (CaCO_3_)/COOH molar ratio of 0.288, and the gelling process was triggered through the addition of GDL in MilliQ water at a CaCO_3_/GDL (Glucono delta-lactone) molar ratio of 0.125. The NaCl particles (0, 0.6, and 1.1 mg/mL) were incorporated into the alginate solution (2 wt.% alginate, 600 μM RGD) and the mixture was poured in a 96-well plate (250 μL/well) for crosslinking. After gelation [≈45 min at room temperature (RT)] samples were frozen at −20°C, and then freeze-dried for 48 h (0.008 mBar, −80°C). The salt particles were then leached out by submerging the scaffolds in distilled water for 24 h with agitation. The leached samples were freeze-dried again for 48 h and the obtained cylindric scaffolds were then cut into discs with an average height of 1.5 mm.

Scaffolds were characterized by Scanning Electron Microscopy (SEM) analysis performed on the surfaces and cross-sections, using a High-resolution Scanning Electron Microscope with X-Ray Microanalysis (JEOL JSM 6301F/Oxford INCA Energy 350) at an accelerating voltage of 15 kV. Samples were coated with Au/Pd thin film, by sputtering for 120 s and with 15 mA current, using the SPI Module Sputter Coater equipment. The average pore diameter was analyzed using Fiji Imaging software.

### Cell Sources and Maintenance

Human outgrowth endothelial cells (OEC) were isolated from umbilical cord blood from a single donor, according to protocols approved by the UC Davis Stem Cell Research Oversight Committee, as reported by Torres and colleagues (Torres et al., 2018). Cells were cultured in endothelial cell growth medium, EGM-2MV (Lonza), prepared by supplementing endothelial basal medium (EBM)-2 with ascorbic acid, hydrocortisone, epidermal growth factor (hEGF), vascular endothelial growth factor (VEGF), basic fibroblast growth factor-b (hFGF-b), insulin growth factor-1 (IGF-1) and 5% v/v fetal bovine serum (FBS). OEC were used between passage 6 and 8.

Human mammary fibroblasts (hMF) were routinely cultured in high glucose Dulbecco’s Modified Eagle Medium GlutaMax^TM^ (Gibco Life Technologies) supplemented with 10% of FBS (Biowest) and 1% of penicillin/streptomycin (Pen/Strep, Sigma). hMF were used between passages 6 and 10.

MCF10A cells (normal-like breast epithelial cell line) were maintained in Dulbecco’s Modified Eagle Medium/Nutrient Mixture F-12 with Glutamax (DMEM/F12 GlutaMAX^TM^, Gibco) supplemented with 5% v/v Horse Serum (Thermo Fisher Scientific), 20 ng/mL EGF (Sigma), 66.6 ng/mL Hydrocortisone (1 mg/mL, Sigma), 100 ng/mL Cholera Toxin (Sigma), 0.01 mg/mL Insulin solution human (Sigma) and 1% v/v Pen/Strep. MCF10A were used between passages 20 and 30.

MCF7 cells (luminal non-metastatic breast cancer cell line) were maintained in phenol red-free DMEM/F12 with Glutamax (Gibco) supplemented with 10% v/v FBS and 1% v/v Pen/Strep. MCF7 were used between passages 14–20.

MDA-MB-231 cells (basal aggressive metastatic breast cancer cell line) were maintained in DMEM with Glutamax (Gibco) supplemented with 10% v/v FBS and 1% v/v Pen/Strep. All cells were kept at 37°C under 5% v/v CO_2_ humidified atmosphere. MDA-MB-231 were used between passages 40–45.

### Monoculture of Fibroblasts or OEC on Porous RGD-Alginate Scaffolds

Sterile RGD-alginate scaffolds were placed in tissue culture well plate and incubated with cell culture medium at 37°C. Fibroblasts or OEC were seeded at 25×10^4^ cells/scaffold in a total volume of 30 μL of the respective culture medium. Two different seeding strategies were tested to select the one promoting better cell distribution throughout the scaffold. In strategy A, 30 μL of cell suspension were added to the top of the scaffold. In strategy B, 15 μL of cell suspension were added on the top and 1 h later the scaffold was turned, and the remaining 15 μL were added on the other side. After allowing cell adhesion for 4 h, culture medium was added, and the seeded scaffolds were incubated. Media was changed every 2 days. For each condition three different biological replicates were performed.

For whole-mounted samples immunostaining, 3D cultures were fixed with 4 wt.% paraformaldehyde (PFA, Sigma) in Tris Buffered Saline Buffer with calcium (TBS-Ca), permeabilized with 0.1% v/v Triton X-100/TBS-Ca and incubated for 1 h in 1 wt.% bovine serum albumin (BSA, Sigma) in TBS-Ca to block unspecific binding. Scaffolds with fibroblasts were incubated overnight (ON) at 4°C with phalloidin 488 (1:40, Flash Phalloidin Green 488, BioLegend) and with rabbit anti-fibronectin (FN, 1:100, Sigma), washed and then incubated with secondary antibody goat anti-rabbit Alexa Fluor 594 (1:1,000) for 1 h.

Scaffolds with OEC were incubated ON at 4°C with rabbit anti-laminin (1:50, Sigma) and mouse anti-CD31 (1:100, clone JC70A, DAKO), washed and then incubated for 1 h with secondary antibodies Alexa Fluor 488 goat anti-rabbit (1:1,000) and Alexa Fluor 594 goat anti-mouse (1:1,000). Nuclei were counterstained with DAPI and samples were imaged by confocal laser scanning microscopy (CLSM, Leica TCS SP5). Data analysis was performed using Fiji Imaging software.

### Co-cultures Fibroblasts and Endothelial Cells on Porous RGD-Alginate Scaffolds

For fibroblasts and OEC co-cultures, sequential and simultaneous seeding approaches were tested. For sequential seeding, fibroblasts were seeded at day 0 at 50×10^4^ cells/scaffold and after 4 or 7 days of culture, 50×10^4^ of OEC were added to the pre-seeded scaffolds. After cell adhesion (4 h), EGM-2MV culture medium was added and scaffolds were incubated. For simultaneous seeding cells were combined at 1:1 ratio, with a final cell density of 50×10^4^ or 100×10^4^ cells/scaffold. The effect of a centrifugation step (5 min, 1,000 rpm) after on-top cell seeding at the lowest density was also tested. Whole-mounted samples were processed for immunostaining and imaged by CLSM as described in the previous section. For each condition two different biological replicates were performed.

### Outward Cell Migration Assay in Fibrin

Alginate scaffolds with fibroblasts and OEC cultured for 7 days were embedded between two layers of fibrin gel (tissue mimic). Briefly, fibrinogen solution (4 mg/mL in 0.9% NaCl, Sigma) supplemented with aprotinin (60 g/mL in PBS, Sigma) was mixed with thrombin (2.1 U/mL in PBS, Sigma) at a 4:5 ratio and placed in a μ-slide 4-well (ibidi^TM^). After 30 min incubation at 37°C, pre-seeded scaffolds were placed on top of the fibrin layer, and then a second layer of fibrin was added. After gel formation EGM-2MV was added and refreshed every day for 3 days. Samples were processed for immunostaining and imaged by CLSM as previously described. Three different biological replicates were performed.

### 3D Culture of Epithelial Cells in RGD-Alginate Hydrogels

For cell entrapment, MCF10A, MCF7, and MDA-MB-231 cells were resuspended at 5 × 10^6^ cells/mL in RGD-alginate solution (200 μM RGD) with crosslinking agents, and hydrogel discs were produced as previously described (Bidarra et al., 2016; Bidarra and Barrias, 2019; Barros da Silva et al., 2020). Briefly, a gel-precursor solution of 1.7 wt.% RGD-alginate in 0.9 wt.% NaCl was sterile-filtered (0.22 μm) and mixed with an aqueous suspension of sterile CaCO_3_ (Fluka) at a CaCO_3_/COOH molar ratio of 1.662. Then, a fresh sterile solution of glucone delta-lactone (GDL, Sigma-Aldrich) was added along with the cells to trigger gelation (final concentration: 1 wt.% alginate, 200 μM RGD). CaCO_3_/GDL molar ratio was set at 0.125 and gelation time was 20 min, to yield soft hydrogels with stiffness around 200 mPA as described in Bidarra et al. (2016). The mixture was pipetted (20 μL) onto Teflon plates separated by 750 μm-height spacers, and after gelation 3D hydrogel matrices were transferred to 24-well culture plates treated with poly(2-hydroxyethyl methacrylate (pHEMA). Thereafter, fresh medium was added and renewed after 1 h. 3D cultures of epithelial cells were maintained in standard medium, which was replenished every other day. For each condition three different biological replicates were performed.

### Epithelial Cells Behavior in 3D Culture

Metabolic activity of epithelial cells embedded in RGD-alginate matrices was determined through a resazurin-based assay. 3D hydrogel matrices were incubated with 20% v/v of stock resazurin solution at 0.1 mg/mL (Sigma) in the respective normal cell culture medium for 2 h at 37°C protected from light. Fluorescence measurements were performed using a microplate reader (Biotek Synergy MX) with excitation/emission at 530/590 nm.

Cell proliferation was analyzed at day 14 of culture, by Ki-67 immunostaining and expression of cell-cell junction markers E-cadherin and β-Catenin. Epithelial-laden hydrogels were fixed with 4 wt.% PFA in TBS-Ca, permeabilized with 0.1% v/v Triton X-100/TBS-Ca and incubated for 1 h in 1 wt.% BSA in TBS-Ca to block unspecific binding. Samples were incubated ON at 4°C with rabbit anti-Ki-67 (1:100, Abcam), followed by goat anti-rabbit secondary antibody Alexa Fluor 488 (Invitrogen, 1:1,000, 1 h at RT). Samples were also incubated ON at 4°C with rabbit anti-E-cadherin (1:100, Cell Signaling) and mouse anti-β-catenin (1:50, BD Bioscience), followed by secondary antibodies Alexa Fluor 488 goat anti-rabbit (Invitrogen, 1:1,000) and Alexa Fluor 594 goat anti-mouse (Invitrogen, 1:1,000) for 1 h at RT. Nuclei were counterstained with DAPI, samples were imaged by CLSM and data analysis was performed using Fiji Imaging software.

### Establishment of a Heterotypic 3D Model With Stromal and Parenchymal Compartments

Epithelial cells were combined with RGD-alginate gel-precursor solutions, as previously described, and added to porous RGD-alginate scaffold previously co-cultured with fibroblast and OEC for 1 week. After 20 min gelation, EGM-2MV media was added, and tri-culture system was maintained in culture for up to 7 days. Samples were processed for immunostaining and imaged by CLSM as previously described. For each condition two different biological replicates were performed.

### Histological Analysis

Whole-mounted samples with both compartments were fixed and embedded in paraffin in an automated tissue processor (Microm STP 210). Sample processing was set to graded series of 20 min each, starting by sequential immersion in ethanol (EtOH) solutions of increasing concentrations (70, 90, 98, and 100%), followed by immersion in ClearRite and finally immersion in preheated paraffin. Samples were embedded in paraffin in a modular embedding system (Microm STP 120-1) with transversal orientation. Paraffin blocks were sectioned (6 μm) using a semi-automated microtome (Leica RM2255). Paraffin-embedded sections were mounted on (3-aminopropyl)triethoxysilane (APES) coated glass slides, dried ON at 37°C and then kept at RT until use. All slides were dewaxed in xylene and dehydrated using an ethanol gradient before stained with safranin-Light Green (SF-LG, for alginate) and hematoxylin (for nuclei). Images were obtained using an inverted fluorescence microscope (Axiovert 200M, Zeiss) and processed using Fiji Imaging Software.

### Statistical Analysis

Statistical analyses were performed using GraphPad Prism 7.0 software version. Normality of data was tested using D’Agostino-Pearson omnibus and Shapiro-wilk tests. For metabolic activity analysis, One-way ANOVA test was used. Results for all analysis with “*p*” value less than 0.05 were considered to indicate statistically significant differences (^∗^*p* < 0.05, ^∗∗^*p* < 0.01 and, ^∗∗∗^*p* < 0.0001).

## Results

### Preparation and Characterization of Anisotropic Porous RGD-Alginate Scaffolds

**FIGURE 2:**
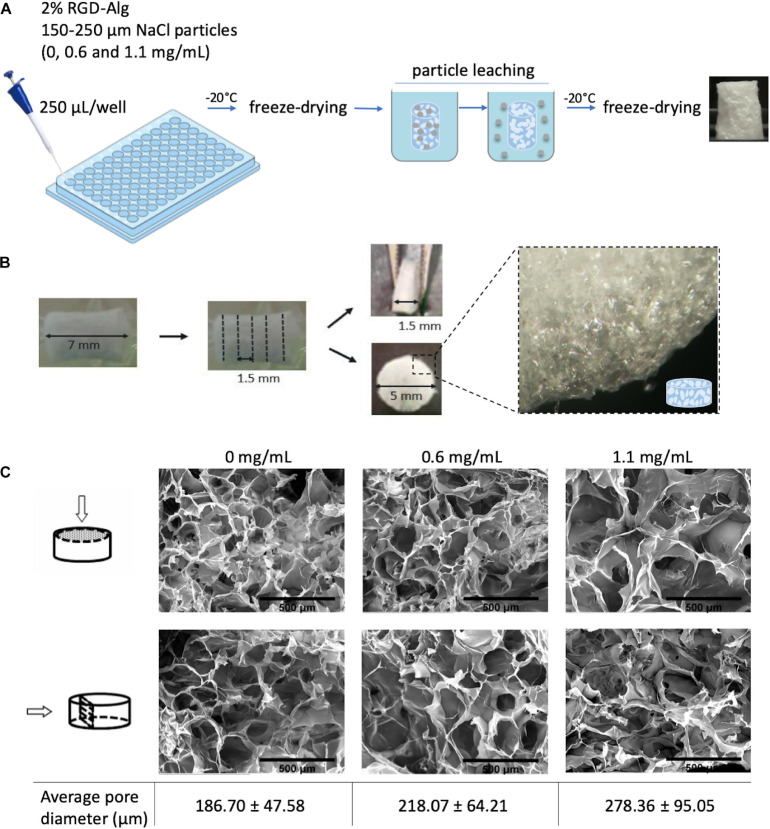
Preparation and characterization of alginate porous scaffolds. **(A)** Schematic representation of freeze-drying combined with particle leaching technique. NaCl particles with particle size of 150–250 μm were incorporated into the alginate precursor solution. After freeze-drying, cylindric scaffolds were placed in distilled water for 24 h with agitation to leach out the salt particles. Leached samples were then freeze-dried again for 48 h. **(B)** Porous cylindric scaffolds were cut into discs with an average height of 1.5 mm. **(C)** SEM images of the surface and cross-section of alginate scaffolds with different pore diameters prepared with different amounts of NaCl particles (0, 0.6, and 1.1 mg/mL); scale bar = 500 μm.

To build a vascularized breast tumor model, a hybrid 3D system was established, consisting of a pre-vascularized porous scaffold (STEP 1) filled with an epithelial cell-laden soft hydrogel (STEP 2) (Figure 1). RGD-modified alginate was selected as an ECM-like matrix for building both the vascularized stromal and parenchymal (epithelial) compartments. Anisotropic microporous alginate scaffolds were produced by combined freeze-drying/particle leaching technique. Sodium chloride (NaCl) was used as porogen and particles with size between 150 and 250 μm were incorporated at different concentrations (0, 0.6, and 1.1. mg/mL) into the 2 wt.% RGD-alginate gel-precursor solution. After crosslinking in 96 well-plates, samples were frozen and freeze-dried, salt particles were leached in distilled water and leached samples were freeze-dried again (Figure 2A). The obtained cylindrical scaffolds were cut into discs with an average height of 1.5 mm (Figure 2B). SEM analysis (Figure 2C) showed that the pores size correlated with the amount of NaCl particles. Higher amounts of NaCl particles resulted on larger pores, reaching a maximum average pore diameter size of ca. 278 μm. The porous structure was interconnected and uniform throughout the scaffold, without significant differences between the surface and inner regions.

### Vascularized Stromal Compartment: Fibroblasts and Endothelial Cells Co-culture in Porous Hydrogel Scaffold

**FIGURE 3:**
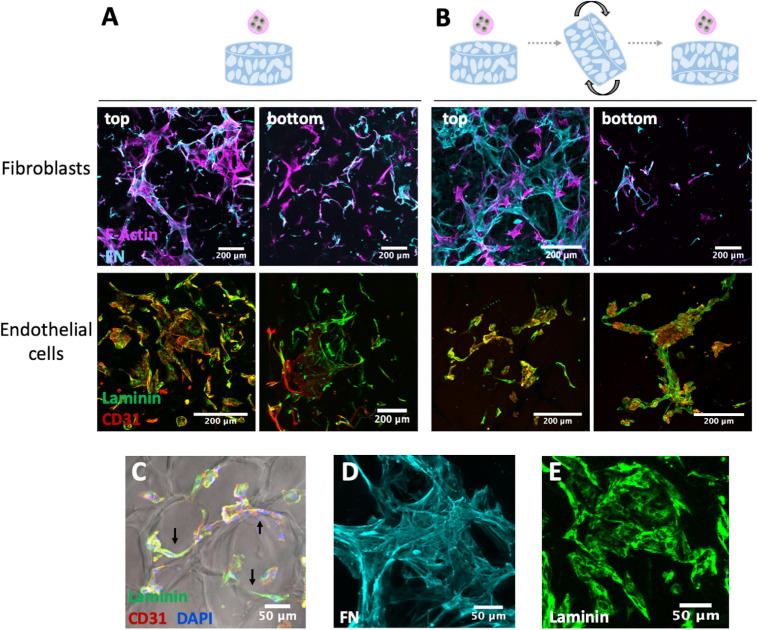
Fibroblasts and OEC cultured on RGD-alginate porous scaffolds after 14 days observed under CLSM using two different seeding approaches: **(A)** only on top, and **(B)** on both sides of the scaffold. Fibroblasts mono-cultures were stained for F-actin (magenta), fibronectin (FN, cyan), and endothelial cells monocultures for laminin (green) and CD31 (red); scale bar = 200 μm. Irrespectively of the approach **(C)** cells were able to attach to the scaffold and align along the pore walls (black arrows); scale bar = 50 μm. Fibroblasts and OEC were able to produce and assemble endogenous ECM proteins, namely **(D)** fibronectin (FN) and **(E)** laminin; scale bar = 50 μm.

To establish the vascular compartment, fibroblasts and OEC were first individually seeded onto 3D porous scaffold and different seeding strategies were tested to achieve a uniform cell colonization and endogenous ECM deposition throughout the scaffolds. In strategy A (Figure 3A), the cell suspension was added only on top of the scaffold, while in strategy B (Figure 3B), cell suspension was added to both sides. Fibroblast behavior was assessed through F-actin staining and fibronectin production, and OEC behavior was analyzed by CD31 (endothelial marker) and laminin staining. Both cell populations were able to adhere and spread on the scaffold, but a higher cell density was observed on top of the scaffold, as compared to the bottom, in both seeding approaches. Some cells aligned along the pores’ walls, as illustrated in Figure 3C, and both cell types were able to produce and build up an endogenous ECM network. Fibroblast showed ability to assemble extensive fibrillar meshes of fibronectin (Figure 3D), a key component of interstitial ECM, and OEC produced laminin (Figure 3E), considered to be the primary determinant of basement membrane assembly. As no major differences were found between the two seeding strategies, in subsequent co-culture studies the cell suspension was added only on top of the scaffold.

For fibroblasts and OEC co-culture, two seeding approaches were tested: sequential and simultaneous seeding. The rationale for seeding cells sequentially (OEC after fibroblasts) was to allow fibroblasts to produce sufficient amounts of ECM, which could potentially support OEC tubulogenesis. In this sense, two timelines were tested, where OEC were seeded 4 or 7 days after fibroblast pre-seeding (Supplementary Figure 1). Immunofluorescence images of both conditions showed that fibroblast pre-seeding led to the formation of a dense monolayer of cells with extensive fibronectin deposition. However, as illustrated by CD31 staining, OEC spread and formed dense monolayers, instead of organizing into tubular-like structures.

**FIGURE 4:**
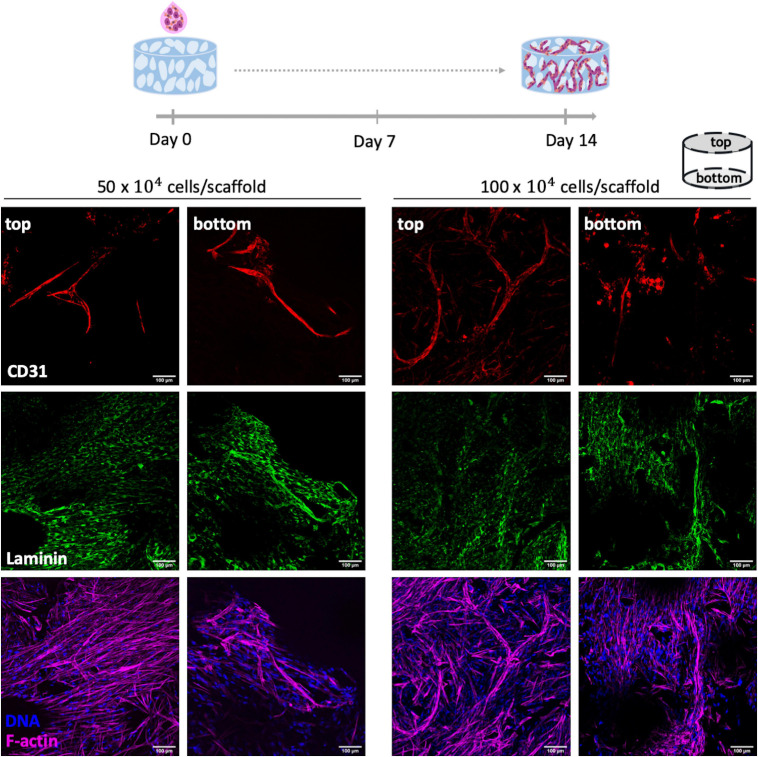
Co-culture of OEC and human mammary fibroblasts on RGD-alginate porous scaffolds. Two different cell densities per scaffold were tested: 50×10^4^ and 100×10^4^. Co-cultures were stained for CD31 (red), laminin (green), F-actin (magenta) and DNA (blue). Cell morphology and ECM deposition on top and bottom of the scaffolds were imaged by CLSM; scale bar = 100 μm.

In the other co-culture strategy, OEC and fibroblast were seeded simultaneously at 1:1 cell ratio, with a total number of cells per scaffold of 50×10^4^ or 100×10^4^ (Figure 4). As depicted in Figure 4 this strategy successfully led to a uniform cell adhesion (F-actin staining) and formation of endothelial tubular-like structures (CD31^+^) with laminin deposition. Noteworthy, cell density did not significantly impact cell organization and, so, the lowest one was selected for subsequent studies.

**FIGURE 5:**
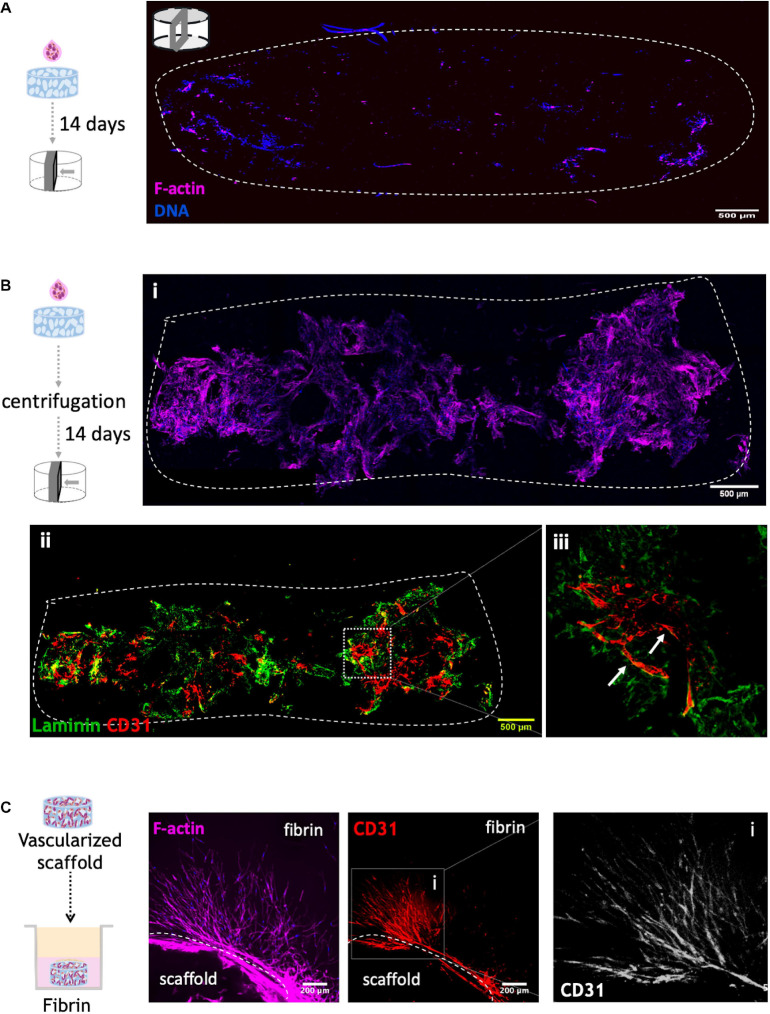
Co-culture of OEC and human mammary fibroblast on RGD-alginate porous scaffolds **(A)** without and **(B)** with centrifugation after adding 50×10^4^ cells on top of the scaffold. Cell distribution was observed under a CLSM by F-actin (magenta) and DNA staining (blue). The dashed white line illustrates the limits of cross-section of the scaffold; scale bar = 500 μm. To assess OEC organization, (ii) centrifuged scaffolds were also stained for CD31 (red) and laminin (green), and (iii) Higher magnification showed OEC organization into tubular-like structures around pores (white arrows). **(C)** Cell outgrowth in fibrin. Co-culture scaffolds cultured for 14 days were transferred to a layer of fibrin gel. Images were obtained by CLSM: F-actin (magenta), CD31 (red) and DNA (blue); scale bar = 200 μm.

In order to further improve cell colonization and infiltration into the scaffold, a centrifugation step was performed immediately after adding the cell suspension to the top of the structure. To evaluate the effect of this step, different F-actin stained cross sections of 14-days cultured scaffolds were analyzed by CLSM (Figures 5A,Bi). As illustrated in Figures 5A,Bi, the centrifugation-based seeding strategy, clearly resulted in the best outcome, with substantial higher amounts of cells throughout the cross sections. Scaffolds were also stained for CD31 and laminin (Figure 5Bii), showing that OEC organized into tubular-like structures in the scaffold core and near the pores (Figure 5Biii). Thus, this combinatory approach was selected for subsequent experiments.

The potential of the vascularized scaffolds to allow cell outgrowth and endothelial tubulogenesis into the external milieu will be key to ensure efficient interaction between the vascular and epithelial compartments after assembling the hybrid system. This was tested by analyzing cell migration/invasion from scaffolds embedded in a tissue-mimic gel (fibrin) (Figure 5C), which showed extensive outward migration of both cell types, with OEC sprouting as tubular-like structures into the fibrin matrix.

### Parenchymal Compartment: Epithelial Cells-Laden *in situ* Forming Hydrogel

**FIGURE 6:**
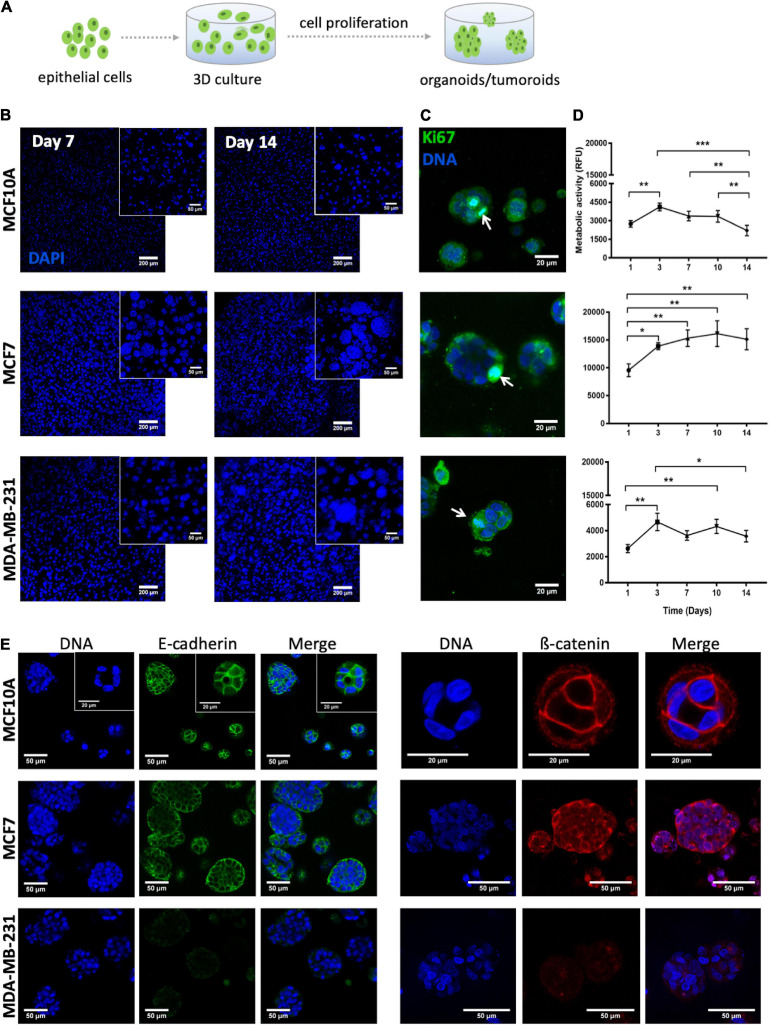
Behavior of MCF10A, MCF7 and MDA-MB-231 inside RGD-alginate 3D hydrogel. **(A)** Schematic representation of epithelial cell embedding. **(B)** Epithelial cell-laden hydrogels after 7 and 14 days of culture formed spheroids that increased both in size and number throughout 14 days of culture. Spheroids were visualized with DAPI staining (blue). Scale bar = 200 μm, inset: 50 μm. **(C)** Proliferating epithelial cells, Ki-67 positive cells (arrows) were detected in spheroids after 14 days of culture. Scale bar = 20 μm. **(D)** Metabolic activity profile in relative fluorescence units (RFU) throughout 14 days of culture. Data is presented as mean ± stdev (*n* = 4) (*p < 0.05, **p < 0.01, and ***p < 0.0001). **(E)** CLSM images of epithelial markers after 14 days of culture: E-cadherin (green) and β-catenin (red). Scale bar = 50 μm, inset = 20 μm.

To build the parenchymal compartment, a previously optimized formulation of soft alginate hydrogels (200 Pa) functionalized with integrin-binding RGD peptides (200 μM) was used to simulate the 3D microenvironment of the normal mammary gland (Bidarra et al., 2016; Barros da Silva et al., 2020). Epithelial cells were combined with a gel precursor solution of RGD-modified alginate and ionic crosslinking agents, becoming entrapped in Ca-alginate hydrogels after gelation (Figure 6A).

To validate the parenchymal compartment, the morphology, metabolic activity and proliferation of three different cell lines (normal mammary epithelial MCF10A cells, non-invasive tumorigenic human breast cancer MCF7 cells, and highly invasive human breast cancer MDA-MB-231 cells) was assessed. In all three cases, cells initially distributed as single cells within the alginate matrix were able to proliferate and grow as multicellular clusters (spheroids), as depicted in Figure 6B. This could already be detected after 7 days, with spheroid size increasing throughout time until day 14. Noteworthy, both MCF7 and MDA-MB-231 cell lines formed larger cell clusters as compared to the non-tumorigenic cell line, MCF10A. The proliferative capacity in 3D was further examined by immunofluorescence using the ki-67 proliferation marker. After 2 weeks of culture, proliferative cells were essentially restricted to spheroids (Figure 6C). MCF7 presented the highest levels of metabolic activity, as compared to MCF10A and MDA-MB-231 (Figure 6D), which gradually increased until day 10. While the metabolic activity profiles of the other two cell lines were less uniform, all of them exhibited a significant rise along the first 3 days of culture, suggesting that cells were actively proliferating with a fold increase similar between all cell lines (Supplementary Figure 2). After day 3, no significant differences were observed, except for MCF10A whose metabolic activity significantly decreased along the second week of culture.

Next, we assessed the presence of cell-cell junction markers in the different epithelial cell lines in 3D. Although all of them formed multicellular clusters, only MCF10A and MCF7 cells expressed E-cadherin and ß-catenin at cell-cell junctions, as depicted in Figure 6E. In MCF10A-laden hydrogels, epithelial morphogenesis into prototypical acini-like structures was observed after 14 days in culture, with an organized layer of single cells oriented around a central lumen (Figure 6E). In contrast, the two tumorigenic cell lines formed denser and less organized multicellular aggregates, recapitulating tumoroid-like structures.

### Hybrid 3D System: Integration of Vascularized Stromal and Parenchymal Compartments

**FIGURE 7:**
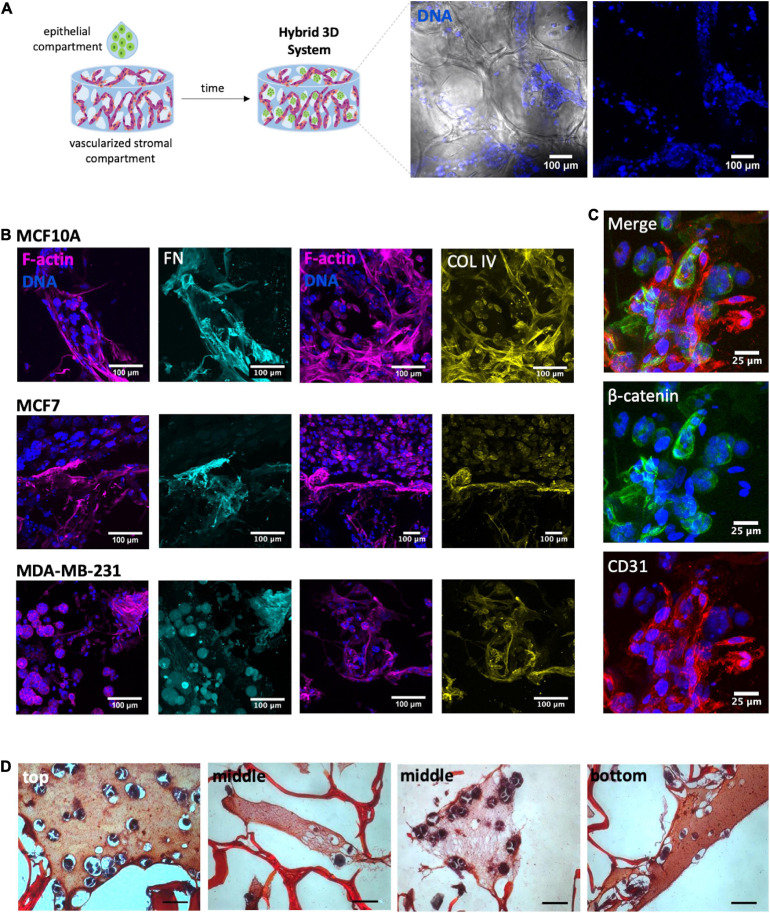
Development of a heterotypic 3D system. **(A)** After 1 week of tri-culture it was possible to observe the presence of spheroids within the porous scaffold: spheroids (nuclei, blue) within the alginate porous (bright field). **(B)** Immunostaining of tri-cultured scaffolds of MCF10A, MCF7 and MDA-MB-231 showed the presence of ECM proteins fibronectin (FN, cyan) and collagen IV (COL IV, yellow). Overall cell organization can be visualized through F-actin (magenta) and nuclei (blue) staining; scale bar = 100 μm. **(C)** In higher magnification images was possible to visualized cell-cell contact between acinar-like structures and OEC (CD31+); scale bar = 25 μm. **(D)** The cross-sections of the constructs with epithelial-laden hydrogels were stained with Safranin, allowing to visualize the presence of spheroids within the structures pores on the top, middle and bottom regions of the scaffold cross-sections; scale bar = 50 μm.

Both the vascularized stromal and the epithelial compartments were finally integrated into a tri-culture hybrid system. Epithelial cells were combined with RGD-alginate gel precursor solution and the mixture was added to the porous of the pre-vascularized scaffolds (pre-cultured for 1 week), forming a hydrogel *in situ* (Figure 7A). CLSM analysis of immunostained scaffolds after 1 week of culture, before adding epithelial cells, confirmed the presence of aligned CD31+ OEC (Supplementary Figure 3), and the deposition of collagen type IV (another major component of the basement membrane) at the periphery of endothelial structures (Supplementary Figure 3A), and fibronectin throughout the scaffold (Supplementary Figure 3B). One week after adding the gel-embedded epithelial cells, it was possible to detect the presence of spheroids within the scaffolds porous, in all the three cell lines (Figure 7B) and in close contact with previously seeded stromal cells and their ECM (Figures 7C,D). To visualize the spatial distribution of spheroids by histological analysis, cross sections of paraffin-embedded 3D constructs were stained with Safranin-Light Green and hematoxylin, which stain alginate in orange/red and cells/tissue in bluish, respectively. This allowed to detect the presence of spheroids on the top, middle and bottom regions of the scaffold cross-sections (Figure 7D).

## Discussion

The stromal microenvironment of breast tumors, namely the vasculature, plays a key role in tumor development and metastatic spread. Whether at the primary site or after metastization, it is known that tumors recruit blood vessels to feed their growth and avoid dormancy (De Palma et al., 2017). Tumor vascularization occurs mainly via sprouting angiogenesis, resulting from aberrant expression of pro-angiogenic factors. This “angiogenic switch” is a complex and dynamic multistep process, involving EC activation, migration, proliferation and organization (Roudsari and West, 2015). The use of 3D tissue engineering models *in vitro* that incorporate not only tumor cells, but also a vascularized structure, certainly help to clarify the molecular/cellular mechanisms involved in the tumor vascularization, the key to unveiling new therapeutic targets. Most of the 3D approaches for tumor angiogenesis engineering make use of ECM-derived hydrogels such as collagen type I and Matrigel^TM^ as 3D matrix, since they carry a variety of intrinsic signals important for tumour morphogenesis, EC capillary morphogenesis and sprouting (Shekhar et al., 2001; Walter-Yohrling et al., 2003; Buchanan et al., 2012, 2014; Correa de Sampaio et al., 2012; Bordeleau et al., 2017). However, major weaknesses of these materials are their inherent variability, limited stiffness range, and structural instability caused by uncontrolled hydrogel degradation and/or shrinkage along culture. Also, cell recovery from these hydrogels requires proteolytic digestion that may negatively impact cells and led to unwanted contamination of samples with protein components. Synthetic materials, such as polyethylene glycol-heparin (PEG) have also been used to develop 3D systems to model tumor angiogenesis (Bray et al., 2015; Taubenberger et al., 2016; Brassard-Jollive et al., 2020). Unlike protein-derived materials, they present controlled composition, along with tunable biochemical/mechanical properties. Also, their intrinsic bio-inertness, coupled with the presentation of functional groups for chemical modification, allows for hydrogel biofunctionalization to specifically control cell response. Yet, crosslinking is irreversible, requiring harsh procedures to recover cells for subsequent analysis. Alginate hydrogels, a well-known natural-derived polysaccharide, combine the advantages of the previous ones. Alginate is bioinert but amenable to all sorts of chemical modification, available in ultra-pure grades, with present low batch-to-batch variability, and their hydrogels present tunable mechanical properties being easily dissolved with chelating agents and/or non-mammalian enzymes. To simultaneously dissolve the hydrogel and efficiently disrupt cell-cell and cell-matrix interactions cellularized alginate hydrogels can be incubated in trypsin/EDTA solution as demonstrated by Barros da Silva et al. (2020).

In this work we selected ultra-pure peptide-modified alginate as the backbone biomaterial for building a hybrid 3D system of vascularized breast, combining gel-embedded epithelial cells with a pre-vascularized 3D porous scaffold co-seeded with fibroblasts and EC. To better replicate breast cancer heterogeneity, three different breast cell lines were used: MDA-MB-231, a highly invasive triple-negative breast cancer cell line, i.e., cells are negative for hormone receptors (estrogen and progesterone) and HER2; MCF-7, a less invasive luminal cell line that is positive for estrogen receptors, but negative for HER2 (Dai et al., 2017); and MCF10A, a non-tumorigenic breast cell line.

To build the vascularized stromal compartment, a porous alginate scaffold was obtained by freeze-drying. This technique is simple, fast, and low-cost and it does not require the use of organic solvents (Loh and Choong, 2013). Furthermore, it allows the formation of pores with sizes that can support the generation of blood vessels since EC migration and metabolites exchanges are guaranteed within pores with a minimum size of 30–40 μm (Loh and Choong, 2013). However, it was also shown that increasing pore sizes to 160–270 μm facilitates new vessels formation throughout the scaffold (Loh and Choong, 2013). Thus, to create structures with larger pores, the particle leaching technique was combined with freeze-drying. The incorporation of NaCl particles between 150 and 250 μm led to the formation of larger and interconnected porous, with an average diameter of 278 μm. Importantly, the porous structure was uniform throughout the scaffold, without significant differences between the surface and the inner regions. To rapidly create large numbers of porous scaffolds a 2 wt. % RGD-alginate precursor gel with NaCl particles was added directly to a 96 well-plate, followed by freeze-drying and particle leaching. The final step of scaffold preparation is freeze-drying, allowing them to be stored and ready-to-use.

To promote pre-vascularization of the porous scaffolds, two different stromal cell types were combined, human OEC and mammary fibroblasts. It is currently well established that EC network formation is enhanced/stabilized in the presence of other stromal cells, like fibroblasts, which not only produce important soluble factors and ECM components, but may also directly contribute to vascular structures maturation via cell-cell interactions (Koike et al., 2004; Torres et al., 2020). OEC present high proliferative capacity and phenotypic stability in long-term monolayer culture (Fuchs et al., 2006). In the proposed model, fibroblasts are expected to play a center microenvironmental role in supporting the angiogenic process, not only through ECM production and remodeling, but also via secretion of angiogenic growth factors such as VEGF and FGF (Newman et al., 2011). Several cell seeding approaches were tested and optimized in order to attain the highest seeding efficiency. Based on previous studies on EC-stromal cells co-culture (Bidarra et al., 2011a; Chen et al., 2012; Torres et al., 2020), the ratio between the two cell types was set at 1:1, but different seeding configurations (sequentially and simultaneously) were tested aiming optimal formation of capillary-like structures. The best outcome was obtained when simultaneously adding both cells on top of the scaffold, followed by brief centrifugation. Dynamic seeding strategies involving the application of external forces are known to improve scaffold colonization (Dar et al., 2002). A centrifugation step immediately after co-seeding, promoted higher cell infiltration and subsequent capillary network formation throughout the scaffold pores. Upon embedding the seeded scaffolds in fibrin, extensive outgrowth cell migration from scaffolds and endothelial tubulogenesis in the fibrin gel were observed, demonstrating the ability of both cell types to migrate/invade and interact with their external milieu.

In the second step of our strategy, we built the parenchymal compartment of the model, using a previously optimized formulation of soft alginate hydrogels functionalized with cell-adhesion RGD peptides (Bidarra et al., 2016; Barros da Silva et al., 2020). We followed the same strategy reported for the entrapment and 3D culture of murine mammary epithelial cells and MCF10A, in which the best results were obtained using 200 μM RGD-modified alginate, a value comparable to that of ECM-derived matrices (Huebsch et al., 2010), and stiffness around 200 Pa as characterized in Bidarra et al. (2016), similar to that of normal mammary tissue (Paszek et al., 2005). As previously demonstrated (Barros da Silva et al., 2020), entrapped MCF10A cells were able to proliferate, forming spheroids that increased in size along the 2 weeks of culture. Some of these multicellular aggregates maturated into organoids with hollow central lumen, resembling mammary gland acini. These breast epithelial organoids expressed prototypical epithelial markers such, as E-cadherin and ß-catenin at the cell membrane, establishing robust cell-cell interactions. In contrast, the two tumorigenic cell lines formed dense and less organized spheroids, resembling epithelial tumoroids. This was expected, as MCF7 and MDA-MB-231, along with other malignant cells, adopt a variety of colony morphologies and share some common aspects such as loss of polarity, disorganized architecture and a failure to arrest growth (Petersen et al., 1992; Cavo et al., 2016, 2018). MDA-MB-231 is a highly invasive cell line that does not express epithelial cell adhesion molecules (Benton et al., 2009), as confirmed herein. On the other hand, MCF7 is a less invasive cell line with high proliferative capacity (Schmid et al., 2020), that typically originates larger cell clusters, but still with tight cell-cell contact. In our model, we were able to successfully culture the three different cell lines. This highlights the potential of soft RGD-alginate to support the development of both healthy and tumoral breast tissue organoids/tumoroids that reflect tumor breast heterogeneity, standing out as an ideal material to build the parenchymal compartment.

The last stage consisted of combining both compartments to create a hybrid tri-culture breast cancer model. Since after 1 week of culture we already had aligned OEC and deposition of type IV collagen and fibronectin in the scaffolds, we selected this time point to add the epithelial cells. These were suspended in alginate gel-precursor solution, which was added to pre-vascularized porous scaffold, before crosslinking. A hydrogel was then formed in-situ, inside the pores. We demonstrated that epithelial cells inside this hydrogel remained able to proliferate, forming multicellular aggregates and undergoing morphogenesis into acini-like organoids and denser tumoroids, as expected. In the hybrid model, the pre-vascularized scaffold becomes fully embedded in the soft epithelial cell-laden hydrogel, and the high surface area provided by the porous structure fosters the interaction between cells in both compartments. Heterotypic cell-cell communication can be both direct, as stromal cells are able to mechanically remodel the soft hydrogel and migrate/invade to a certain extent reaching the epithelial structures (Maia et al., 2014), and indirect (paracrine), as soluble mediators may permeate the hydrogel network. In future studies, direct cell-cell interactions can be further promoted by using protease-sensitive alginate hydrogels, as previously described by our group (Fonseca et al., 2011, 2013, 2014). Such hydrogels support cell-driven enzymatic matrix remodeling, thus facilitating cellular activity/mobility inside the hydrogel.

Notably, alginate hydrogels can be maintained in culture for long periods of time without losing structural integrity, as demonstrated in several previous studies (Bidarra et al., 2010, 2011b, 2016; Maia et al., 2014; Torres et al., 2018, 2020; Barros da Silva et al., 2020). Moreover, it can be easily dissolved with a chelating agent or alginate-specific enzyme, without impairing cell viability, as previously described (Bidarra et al., 2011b, 2016; Torres et al., 2018, 2020; Barros da Silva et al., 2020). This is key for the detailed characterization of isolated cell populations after co-culture. In future studies, we intend to evaluate how vascular EC genetic profile is altered during and after anti-angiogenic therapy or in the presence of tumoroids with different metastatic capacity in a 3D microenvironment.

## Conclusion

Taken together, our results demonstrate the successful establishment of a hybrid 3D tumor angiogenesis model, with both vascularized stromal and parenchymal compartments. Endothelial and epithelial cells and fibroblasts share the same microenvironment, establishing direct and indirect cell-cell communication. The use of 3D tumor angiogenesis models to study the dynamics of EC recruitment and growth of new vessels toward the tumor mass can greatly improve our understanding of tumor-driven angiogenesis, leading to the development of more effective therapeutic strategies to selectively fight cancer. In the future, this platform can be further adapted to incorporate patient-derived cells for *ex vivo* assessment of therapeutic efficacy using precision medicine approaches for translational research.

## Data Availability Statement

The raw data supporting the conclusions of this article will be made available by the authors, without undue reservation.

## Author Contributions

FT and SC designed the experiments, acquired, and analyzed the data. AT helped in the designed experiments related to endothelial 3D cell culture. CB and SB designed and supervised the whole work, analyzed the data and wrote/revised the manuscript. All authors contributed to the article and approved the submitted version.

## Conflict of Interest

The handling editor declared past co-authorship with one of the authors CB. The remaining authors declare that the research was conducted in the absence of any commercial or financial relationships that could be construed as a potential conflict of interest.

## References

[B1] Araujo M. Bidarra S. J. Alves P. M. Valcarcel J. Vazquez J. A. Barrias C. C. (2020). Coumarin-grafted blue-emitting fluorescent alginate as a potentially valuable tool for biomedical applications. *J. Mater. Chem. B* 8 813–825. 10.1039/c9tb01402k 31909410

[B2] Baliu-Pique M. Pandiella A. Ocana A. (2020). Breast cancer heterogeneity and response to novel therapeutics. *Cancers (Basel)* 12:3271. 10.3390/cancers12113271 33167363 PMC7694303

[B3] Barros da Silva P. Coelho M. Bidarra S. J. Neves S. C. Barrias C. C. (2020). Reshaping in vitro models of breast tissue: integration of stromal and parenchymal compartments in 3D printed hydrogels. *Front. Bioeng. Biotechnol.* 8:494. 10.3389/fbioe.2020.00494 32596217 PMC7300215

[B4] Belgodere J. A. King C. T. Bursavich J. B. Burow M. E. Martin E. C. Jung J. P. (2018). Engineering breast cancer microenvironments and 3D bioprinting. *Front. Bioeng. Biotechnol.* 6:66. 10.3389/fbioe.2018.00066 29881724 PMC5978274

[B5] Benton G. Crooke E. George J. (2009). Laminin-1 induces E-cadherin expression in 3-dimensional cultured breast cancer cells by inhibiting DNA methyltransferase 1 and reversing promoter methylation status. *FASEB J* 23 3884–3895. 10.1096/fj.08-128702 19635753

[B6] Bidarra S. J. Barrias C. C. (2019). 3D culture of mesenchymal stem cells in alginate hydrogels. *Methods Mol. Biol.* 2002 165–180. 10.1007/7651_2018_185 30244438

[B7] Bidarra S. J. Barrias C. C. Barbosa M. A. Soares R. Amédée J. Granja P. L. (2011a). Phenotypic and proliferative modulation of human mesenchymal stem cells via crosstalk with endothelial cells. *Stem Cell Res.* 7 186–197. 10.1016/j.scr.2011.05.006 21907162

[B8] Bidarra S. J. Barrias C. C. Barbosa M. A. Soares R. Granja P. L. (2010). Immobilization of human mesenchymal stem cells within RGD-grafted alginate microspheres and assessment of their angiogenic potential. *Biomacromolecules* 11 1956–1964. 10.1021/bm100264a 20690708

[B9] Bidarra S. J. Barrias C. C. Fonseca K. B. Barbosa M. A. Soares R. A. Granja P. L. (2011b). Injectable in situ crosslinkable RGD-modified alginate matrix for endothelial cells delivery. *Biomaterials* 32 7897–7904. 10.1016/j.biomaterials.2011.07.013 21784515

[B10] Bidarra S. J. Oliveira P. Rocha S. Saraiva D. P. Oliveira C. Barrias C. C. (2016). A 3D in vitro model to explore the inter-conversion between epithelial and mesenchymal states during EMT and its reversion. *Sci. Rep.* 6:27072. 10.1038/srep27072 27255191 PMC4891772

[B11] Bordeleau F. Mason B. N. Lollis E. M. Mazzola M. Zanotelli M. R. Somasegar S. (2017). Matrix stiffening promotes a tumor vasculature phenotype. *Proc. Natl. Acad. Sci. U. S. A.* 114 492–497. 10.1073/pnas.1613855114 28034921 PMC5255592

[B12] Brassard-Jollive N. Monnot C. Muller L. Germain S. (2020). In vitro 3D systems to model tumor angiogenesis and interactions with stromal cells. *Front. Cell Dev. Biol.* 8:594903. 10.3389/fcell.2020.594903 33224956 PMC7674638

[B13] Bray L. J. Binner M. Holzheu A. Friedrichs J. Freudenberg U. Hutmacher D. W. (2015). Multi-parametric hydrogels support 3D in vitro bioengineered microenvironment models of tumour angiogenesis. *Biomaterials* 53 609–620. 10.1016/j.biomaterials.2015.02.124 25890757

[B14] Buchanan C. F. Szot C. S. Wilson T. D. Akman S. Metheny-Barlow L. J. Robertson J. L. (2012). Cross-talk between endothelial and breast cancer cells regulates reciprocal expression of angiogenic factors in vitro. *J. Cell. Biochem.* 113 1142–1151. 10.1002/jcb.23447 22095586

[B15] Buchanan C. F. Voigt E. E. Szot C. S. Freeman J. W. Vlachos P. P. Rylander M. N. (2014). Three-dimensional microfluidic collagen hydrogels for investigating flow-mediated tumor-endothelial signaling and vascular organization. *Tissue Eng. Part C Methods* 20 64–75. 10.1089/ten.TEC.2012.0731 23730946 PMC3870485

[B16] Campiglio C. E. Bidarra S. J. Draghi L. Barrias C. C. (2020). Bottom-up engineering of cell-laden hydrogel microfibrous patch for guided tissue regeneration. *Mater. Sci. Eng. C Mater. Biol. Appl.* 108:110488. 10.1016/j.msec.2019.110488 31924002

[B17] Cavo M. Caria M. Pulsoni I. Beltrame F. Fato M. Scaglione S. (2018). A new cell-laden 3D Alginate-Matrigel hydrogel resembles human breast cancer cell malignant morphology, spread and invasion capability observed “in vivo”. *Sci. Rep.* 8:5333. 10.1038/s41598-018-23250-4 29593247 PMC5871779

[B18] Cavo M. Fato M. Penuela L. Beltrame F. Raiteri R. Scaglione S. (2016). Microenvironment complexity and matrix stiffness regulate breast cancer cell activity in a 3D in vitro model. *Sci. Rep.* 6:35367. 10.1038/srep35367 27734939 PMC5062115

[B19] Chen Y.-C. Lin R.-Z. Qi H. Yang Y. Bae H. Melero-Martin J. M. (2012). Functional human vascular network generated in photocrosslinkable gelatin methacrylate hydrogels. *Adv. Funct. Mater.* 22 2027–2039. 10.1002/adfm.201101662 22907987 PMC3422083

[B20] Coates A. S. Winer E. P. Goldhirsch A. Gelber R. D. Gnant M. Piccart-Gebhart M. (2015). Tailoring therapies–improving the management of early breast cancer: St Gallen international expert consensus on the primary therapy of early breast cancer 2015. *Ann. Oncol.* 26 1533–1546. 10.1093/annonc/mdv221 25939896 PMC4511219

[B21] Correa de Sampaio P. Auslaender D. Krubasik D. Failla A. V. Skepper J. N. Murphy G. (2012). A heterogeneous in vitro three dimensional model of tumour-stroma interactions regulating sprouting angiogenesis. *PLoS One* 7:e30753. 10.1371/journal.pone.0030753 22363483 PMC3282728

[B22] Dai X. Cheng H. Bai Z. Li J. (2017). Breast cancer cell line classification and its relevance with breast tumor subtyping. *J. Cancer* 8 3131–3141. 10.7150/jca.18457 29158785 PMC5665029

[B23] Dar A. Shachar M. Leor J. Cohen S. (2002). Optimization of cardiac cell seeding and distribution in 3D porous alginate scaffolds. *Biotechnol. Bioeng.* 80 305–312. 10.1002/bit.10372 12226863

[B24] De Palma M. Biziato D. Petrova T. V. (2017). Microenvironmental regulation of tumour angiogenesis. *Nat. Rev. Cancer* 17 457–474. 10.1038/nrc.2017.51 28706266

[B25] Drost J. Clevers H. (2018). Organoids in cancer research. *Nat. Rev. Cancer* 18 407–418. 10.1038/s41568-018-0007-6 29692415

[B26] Fonseca K. Bidarra S. J. Oliveira M. J. Granja P. L. Barrias C. C. (2011). Molecularly-designed alginate hydrogels susceptible to local proteolysis as 3D cellular microenvironments. *Acta Biomater.* 7 1674–1682. 10.1016/j.actbio.2010.12.029 21193068

[B27] Fonseca K. B. Gomes D. B. Lee K. Santos S. G. Sousa A. Silva E. A. (2014). Injectable MMP-sensitive alginate hydrogels as hMSC delivery systems. *Biomacromolecules* 15 380–390. 10.1021/bm4016495 24345197 PMC3918418

[B28] Fonseca K. B. Maia F. R. Cuz F. A. Andrade D. Juliano M. A. Granja P. L. (2013). Enzymatic, physiocochemical and biological properties of MMP-sensitive alginate hydrogels. *Soft Matter* 9 3283–3292. 10.1039/c3sm27560d

[B29] Fuchs S. Hermanns M. I. Kirkpatrick C. J. (2006). Retention of a differentiated endothelial phenotype by outgrowth endothelial cells isolated from human peripheral blood and expanded in long-term cultures. *Cell Tissue Res.* 326 79–92. 10.1007/s00441-006-0222-4 16736194

[B30] Gill B. J. West J. L. (2014). Modeling the tumor extracellular matrix: tissue engineering tools repurposed towards new frontiers in cancer biology. *J. Biomech.* 47 1969–1978. 10.1016/j.jbiomech.2013.09.029 24300038

[B31] Grebenyuk S. Ranga A. (2019). Engineering organoid vascularization. *Front. Bioeng. Biotechnol.* 7:39. 10.3389/fbioe.2019.00039 30941347 PMC6433749

[B32] Huebsch N. Arany P. R. Mao A. S. Shvartsman D. Ali O. A. Bencherif S. A. (2010). Harnessing traction-mediated manipulation of the cell-matrix interface to control stem cell fate. *Nat. Mater.* 9 518–526. 10.1038/nmat2732 20418863 PMC2919753

[B33] Koike N. Fukumura D. Gralla O. Au P. Schechner J. S. Jain R. K. (2004). Creation of long-lasting blood vessels. *Nature* 428 138–139. 10.1038/428138a 15014486

[B34] Langhans S. A. (2018). Three-dimensional in vitro cell culture models in drug discovery and drug repositioning. *Front. Pharmacol.* 9:6. 10.3389/fphar.2018.00006 29410625 PMC5787088

[B35] Loh Q. L. Choong C. (2013). Three-dimensional scaffolds for tissue engineering applications: role of porosity and pore size. *Tissue Eng. Part B Rev.* 19 485–502. 10.1089/ten.TEB.2012.0437 23672709 PMC3826579

[B36] Maia F. R. Fonseca K. B. Rodrigues G. Granja P. L. Barrias C. C. (2014). Matrix-driven formation of mesenchymal stem cell-extracellular matrix microtissues on soft alginate hydrogels. *Acta Biomater.* 10 3197–3208. 10.1016/j.actbio.2014.02.049 24607421

[B37] Monteiro M. V. Gaspar V. M. Ferreira L. P. Mano J. F. (2020). Hydrogel 3D in vitro tumor models for screening cell aggregation mediated drug response. *Biomater. Sci.* 8 1855–1864. 10.1039/c9bm02075f 32091033

[B38] Newman A. C. Nakatsu M. N. Chou W. Gershon P. D. Hughes C. C. W. Adams J. C. (2011). The requirement for fibroblasts in angiogenesis: fibroblast-derived matrix proteins are essential for endothelial cell lumen formation. *Mol. Biol. Cell* 22 3791–3800. 10.1091/mbc.e11-05-0393 21865599 PMC3192859

[B39] Papalazarou V. Salmeron-Sanchez M. Machesky L. M. (2018). Tissue engineering the cancer microenvironment-challenges and opportunities. *Biophys. Rev.* 10 1695–1711. 10.1007/s12551-018-0466-8 30406572 PMC6297082

[B40] Pape J. Magdeldin T. Ali M. Walsh C. Lythgoe M. Emberton M. (2019). Cancer invasion regulates vascular complexity in a three-dimensional biomimetic model. *Eur. J. Cancer* 119 179–193. 10.1016/j.ejca.2019.07.005 31470251

[B41] Park K. M. Lewis D. Gerecht S. (2017). Bioinspired hydrogels to engineer cancer microenvironments. *Annu. Rev. Biomed. Eng.* 19 109–133. 10.1146/annurev-bioeng-071516-044619 28633560 PMC5784262

[B42] Paszek M. J. Zahir N. Johnson K. R. Lakins J. N. Rozenberg G. I. Gefen A. (2005). Tensional homeostasis and the malignant phenotype. *Cancer Cell* 8 241–254. 10.1016/j.ccr.2005.08.010 16169468

[B43] Petersen O. W. Ronnov-Jessen L. Howlett A. R. Bissel M. J. (1992). Interaction with basement membrane serves to rapidly distinguish growth and differentiation pattern of normal and malignant human breast epithelial cells. *Proc. Natl Acad. Sci. U. S. A.* 89 9064–9068. 1384042 10.1073/pnas.89.19.9064 PMC50065

[B44] Roudsari L. C. West J. L. (2015). Studying the influence of angiogenesis in in vitro cancer model systems. *Adv. Drug Deliv. Rev.* 97 250–259. 10.1016/j.addr.2015.11.004 26571106

[B45] Schmid R. Schmidt S. K. Hazur J. Detsch R. Maurer E. Boccaccini A. R. (2020). Comparison of hydrogels for the development of well-defined 3D cancer models of breast cancer and melanoma. *Cancers* 12:2320. 10.3390/cancers12082320 32824576 PMC7465483

[B46] Shekhar M. P. V. Werdell J. Santner S. J. Pauley R. J. Tait L. (2001). Breast stroma plays a dominant regulatory role in breast epithelial growth and differentiation- implications for tumor development and progression. *Cancer Res.* 61 1320–1326. 11245428

[B47] Taubenberger A. V. Bray L. J. Haller B. Shaposhnykov A. Binner M. Freudenberg U. (2016). 3D extracellular matrix interactions modulate tumour cell growth, invasion and angiogenesis in engineered tumour microenvironments. *Acta Biomater.* 36 73–85. 10.1016/j.actbio.2016.03.017 26971667

[B48] Thomas D. W. Burns J. Audette J. Carroll A. Dow-Hygelund C. Hay M. (2016). Clinical development success rates 2006–2015. *BIO Ind. Analysis* 1:16.

[B49] Torres A. L. Bidarra S. J. Pinto M. T. Aguiar P. C. Silva E. A. Barrias C. C. (2018). Guiding morphogenesis in cell-instructive microgels for therapeutic angiogenesis. *Biomaterials* 154 34–47. 10.1016/j.biomaterials.2017.10.051 29120817

[B50] Torres A. L. Bidarra S. J. Vasconcelos D. P. Barbosa J. N. Silva E. A. Nascimento D. S. (2020). Microvascular engineering: dynamic changes in microgel-entrapped vascular cells correlates with higher vasculogenic/angiogenic potential. *Biomaterials* 228:119554. 10.1016/j.biomaterials.2019.119554 31677395

[B51] Walter-Yohrling J. Pratt B. M. Ledbetter S. Teicher B. A. (2003). Myofibroblasts enable invasion of endothelial cells into three-dimensional tumor cell clusters: a novel in vitro tumor model. *Cancer Chemother. Pharmacol.* 52 263–269. 10.1007/s00280-003-0664-2 12879277

