# Supplementary material for: Engineering a Vascularized 3D Hybrid System to Model Tumor-Stroma Interactions in Breast Cancer
Source: Front Bioeng Biotechnol. 2021 Mar 11;9:647031. doi: 10.3389/fbioe.2021.647031 (PMC8006407; doi:10.3389/fbioe.2021.647031)
Supplement: Supplementary file 1 [file Data_Sheet_1.DOCX]

Supplementary Material

## Supplementary Figure 1


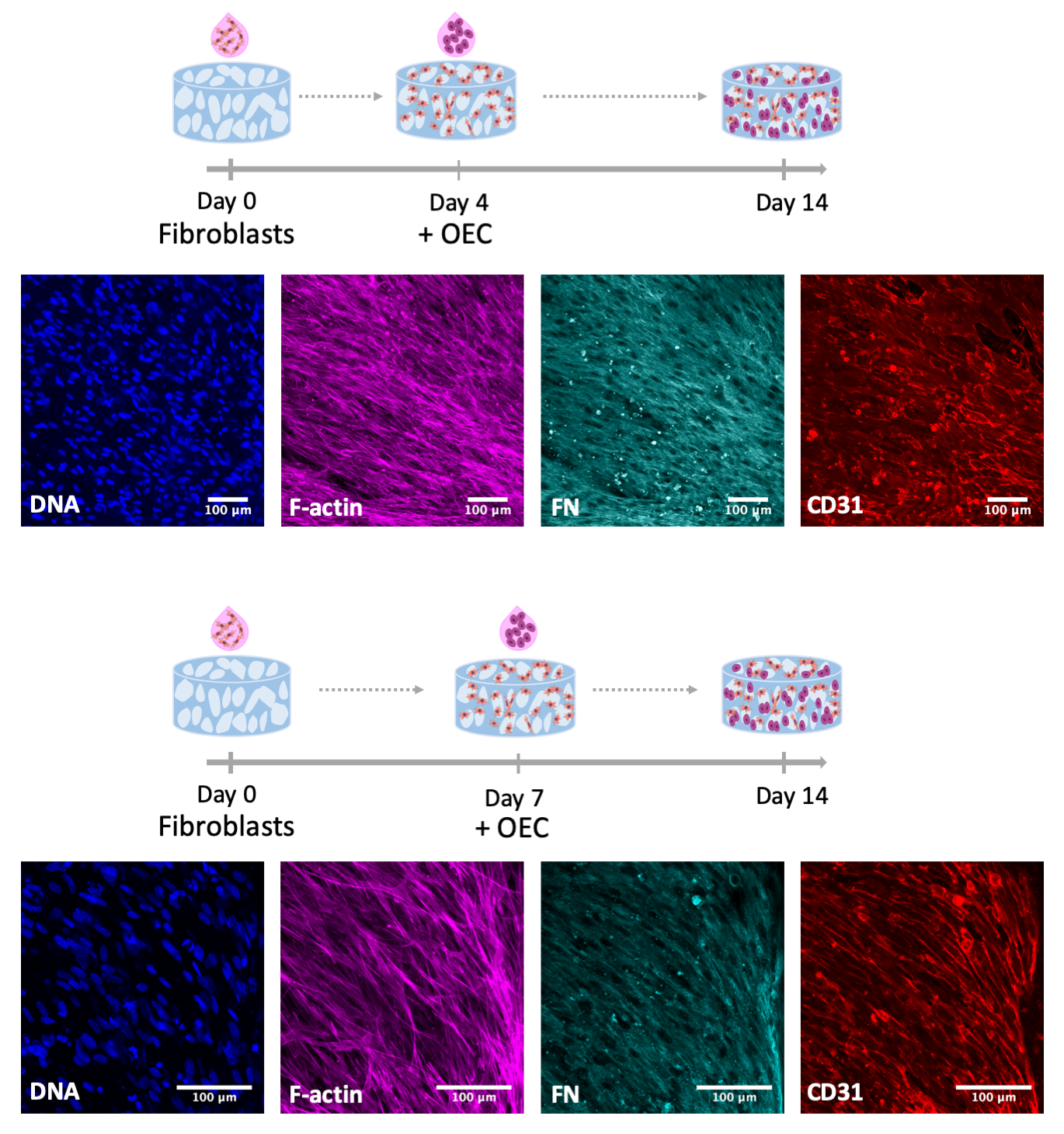


**Supplementary Figure 1.** Co-culture of outgrowth endothelial cells (OEC) and human mammary fibroblasts on RGD-alginate porous scaffolds. Two timelines were tested, where OEC were seeded at day 4 or 7 after fibroblast pre-seeding. Cell morphology  and ECM deposition were imaged by CLSM, revealing no OEC alignment into tubular-like structures. DNA (blue), F-actin (magenta), fibronectin (cyan) and CD31 (red).  Scale bar = 100 µm.
