# Supplementary material for: Engineering a Vascularized 3D Hybrid System to Model Tumor-Stroma Interactions in Breast Cancer
Source: Front Bioeng Biotechnol. 2021 Mar 11;9:647031. doi: 10.3389/fbioe.2021.647031 (PMC8006407; doi:10.3389/fbioe.2021.647031)
Supplement: Supplementary file 2 [file Data_Sheet_2.DOCX]

Supplementary Material

## Supplementary Figure 2


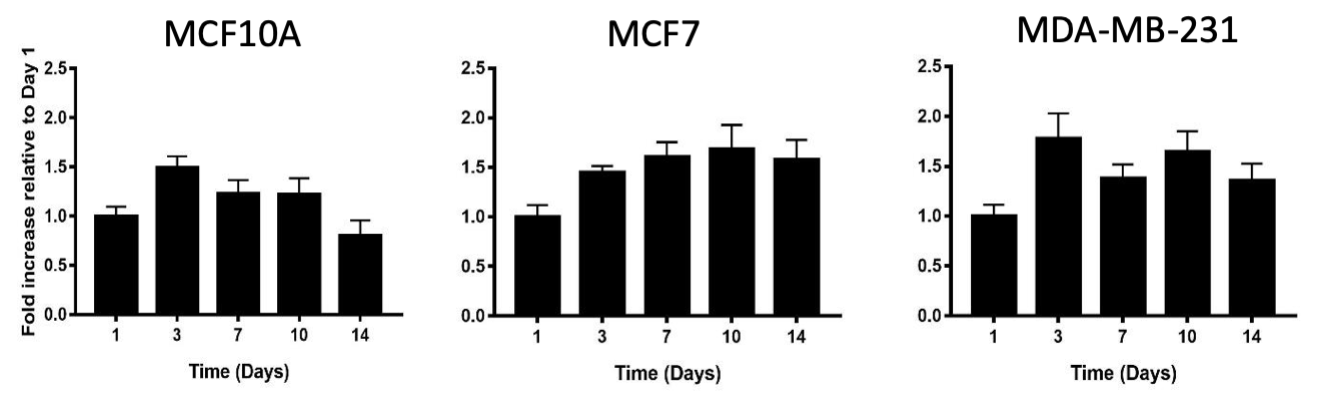


**Supplementary Figure 2.** Fold increase of metabolic activity relative to day 1 of MCF10A, MCF7 and MDA-MB-231 cells within 3D RGD-alginate hydrogel during 14 days of culture. Data is presented as mean ± stdev (n=4)
