# Supplementary material for: Engineering a Vascularized 3D Hybrid System to Model Tumor-Stroma Interactions in Breast Cancer
Source: Front Bioeng Biotechnol. 2021 Mar 11;9:647031. doi: 10.3389/fbioe.2021.647031 (PMC8006407; doi:10.3389/fbioe.2021.647031)
Supplement: Supplementary file 3 [file Data_Sheet_3.DOCX]

Supplementary Material

## Supplementary Figure 3


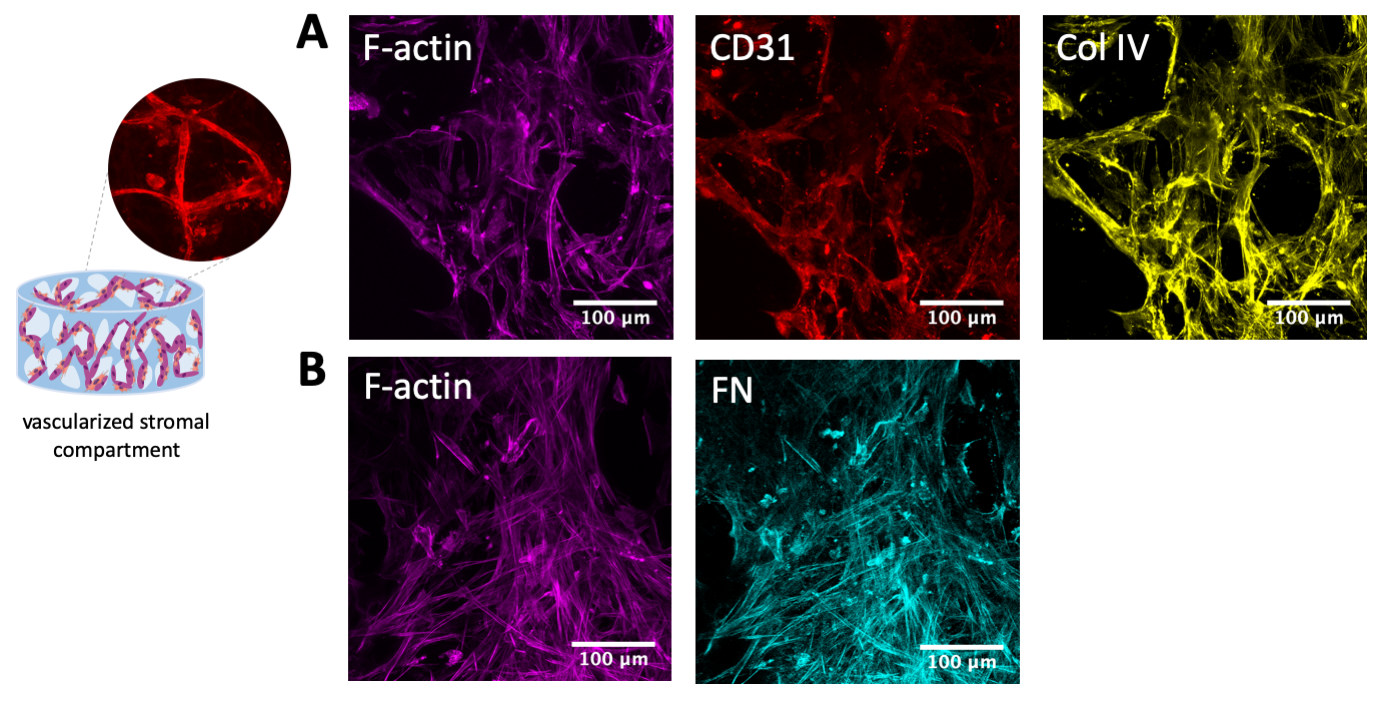


**Supplementary Figure 3.** CLSM images of RGD-alginate scaffolds colonized with outgrowth endothelial cells and human mammary fibroblast for 8 days. Immunostaining of co-cultured scaffolds confirmed the presence of (A) aligned CD31+ OEC (red), deposition of collagen IV at the periphery of endothelial structures (Col IV, yellow), and (B) deposition of fibronectin throughout the scaffold (FN, cyan). Overall cell organization can be visualized through F-actin (magenta) staining. Scale bar: 100 μm.
